# Supplementary material for: Empirical methods for controlling false positives and estimating confidence in ChIP-Seq peaks
Source: BMC Bioinformatics. 2008 Dec 5;9:523. doi: 10.1186/1471-2105-9-523 (PMC2628906; doi:10.1186/1471-2105-9-523)
Supplement: Additional File 1 — A variety of html documents from the USeq web site detailing the available applications, their best usage, output file type descriptions, command line menus, etc. [file 1471-2105-9-523-S1.zip › USeqUserGuides/JavaDoc.zip!/JavaDoc/selex/SubSeq.html]

SubSeq


|  |  |  |
| --- | --- | --- |
| **Overview** \| **Package** \| **Class** \| **Use** \| **Tree** \| **Deprecated** \| **Index** \| **Help** | |  |
| **PREV CLASS**   **NEXT CLASS** | **FRAMES**    **NO FRAMES**     **All Classes** |  |
| SUMMARY: NESTED \| FIELD \| CONSTR \| METHOD | DETAIL: FIELD \| CONSTR \| METHOD |  |


---


## selex Class SubSeq

```
java.lang.Object
  selex.SubSeq
```

---

``` public class SubSeq extends java.lang.Object ```

Holds information related to a particular parsed SELEX oligo.

---

**Constructor Summary**

`SubSeq(java.lang.String sequence,
int[] qualityScores,
SelexParams SP,
SeqRead srObjectRef)`


| **Method Summary** | |
| --- | --- |
| `int` | `getLength()` |
| `int[]` | `getQualScores()` |
| `java.lang.String` | `getSeq()` |
| `SeqRead` | `getSeqRead()` |
| `java.lang.String` | `getSubSeq()` |
| `void` | `printSubSeq()` |

**Methods inherited from class java.lang.Object**

`equals, getClass, hashCode, notify, notifyAll, toString, wait, wait, wait`

**Constructor Detail**

### SubSeq

```
public SubSeq(java.lang.String sequence,
              int[] qualityScores,
              SelexParams SP,
              SeqRead srObjectRef)
```


**Method Detail**

### getSeq

```
public java.lang.String getSeq()
```

---


### getSubSeq

```
public java.lang.String getSubSeq()
```

---


### getLength

```
public int getLength()
```

---


### getQualScores

```
public int[] getQualScores()
```

---


### getSeqRead

```
public SeqRead getSeqRead()
```

---


### printSubSeq

```
public void printSubSeq()
```


---


|  |  |  |
| --- | --- | --- |
| **Overview** \| **Package** \| **Class** \| **Use** \| **Tree** \| **Deprecated** \| **Index** \| **Help** | |  |
| **PREV CLASS**   **NEXT CLASS** | **FRAMES**    **NO FRAMES**     **All Classes** |  |
| SUMMARY: NESTED \| FIELD \| CONSTR \| METHOD | DETAIL: FIELD \| CONSTR \| METHOD |  |


---
